# Where Is the Limit to Christmas Festivities

**Published:** 1914-01-17

**Authors:** James Henry Smethurst

**Affiliations:** Chairman, Warrington Infirmary


*To the Editor of* THE HOSPITAL.

### Where is the Limit to Christmas Festivities?

*To the Editor of* THE HOSPITAL.

SIR,—The query of the secretary of a large London hospital in your last issue as to Christmas entertainment for the staff inclines me again to submit for the consideration of your readers ideas which may probably give some little food for thought and possibly be of benefit. Personally, during the past few years I have tried to arrive at a satisfactory solution of what is, as the secretary states, a difficult problem.

In the first place, every consideration must be given to the patients themselves, who most certainly would not, if they could help themselves, be away from their relations and friends at the happiest time of the year, and I know only too well the feelings of sadness that creep over their minds when they think of their position, and it behoves the managers of every hospital to try and make the time as happy as possible. One ought to banish from the wards every sign that the institution is a charity, and I have always seen to it that collecting boxes are conspicuous by their absence for at all events one week in the year.

Then no part of the expense should be borne either by the general funds or the staff themselves, and it is surprising the difference it makes to a sister in charge of a ward when she knows that any reasonable expenditure will be met by the special fund. . . .

Our Christmas-tree this year was again a most delightful experience, and it enabled us to invite very many people to witness the proceedings, and without a doubt increased their interest in the work. Again, we ought to consider, in my opinion, the children in the Home and out-patient departments. This year we had nearly 400 on the books under eight years of age, and had to hire a large drill-hall to give them tea and presents. In this department it is most desirable to enlist the services of the visiting doctors, who know the actual circumstances of the patients, then cater accordingly. I consider that the presents given should be such as the responsible persons would not be ashamed to give to their own friends and acquaintances.

Then, dealing with the staff, one recognises that week in and week out their life is, at the best, not an easy one, and certainly every member ought to be remembered on Christmas Day and at the Christmas-tree.

Can your readers picture the delight of the smallest ward-maid on receiving her present from some prominent local lady, and the cheers which greet an especially popular nurse when she is called to receive her gift? This, however, in my opinion, is not sufficient, and arrangements ought to be made for the staff to have a special entertainment entirely as guests. I have tried a staff dance several years, but there are disadvantages in this, as often some members do not dance or are strangers in the district, and possibly—as in our case now—the suitable accommodation is too limited, and therefore arrangements have been made for the nursing staff to attend in parties a Manchester theatre of their own selection, and all expenses are paid. Every member of the general staff had their Christmas dinner, and received two tickets for a local theatre and a box of chocolates. . . .

Some of your readers will no doubt say that this costs a lot of money, but I find that the expenditure is one of the best investments the authorities make, as it proves that they are not asking every time for money, but will do a little for their supporters sometimes. One can always obtain gifts of toys and clothing, which come in most useful.

Last year I sent you an account of how I got the money. This year a similar method was adopted, and I am delighted to think that what with subscriptions of £41 9s. 7d. and my week on the stages of the various places of amusement—£76 16s. 4d.—I broke last year's record and have a surplus of over £69 to devote to certain extraordinary expenditure, and in addition give a donation to our Samaritan and Convalescents Funds.

There were over sixty in-patients, a staff of fifty, and, as I have already mentioned, nearly 400 out-patient children. In conclusion, I can assure your readers that it is more than recompensed when I consider that the reputation of our local hospital is higher than ever it was, our staff contented and most willing to do whatever is asked of them.—Yours faithfully,

JAMES HENRY SMETHURST,
Chairman, Warrington Infirmary.

